# Sijilli: A Scalable Model of Cloud-Based Electronic Health Records for Migrating Populations in Low-Resource Settings

**DOI:** 10.2196/18183

**Published:** 2020-08-13

**Authors:** Shadi Saleh, Nour El Arnaout, Lina Abdouni, Zeinab Jammoul, Noha Hachach, Amlan Dasgupta

**Affiliations:** 1 Global Health Institute American University of Beirut Beirut Lebanon; 2 Department of Health Management and Policy Faculty of Health Sciences American University of Beirut Beirut Lebanon; 3 American University of Beirut Medical Center Beirut Lebanon; 4 Epic Systems Corporation Verona, WI United States

**Keywords:** eHealth, digital health, innovation, refugees, low- and middle-income countries, technology

## Abstract

The world is witnessing an alarming rate of displacement and migration, with more than 70.8 million forcibly displaced individuals, including 26 million refugees. These populations are known to have increased vulnerability and susceptibility to mental and physical health problems due to the migration journey. Access of these individuals to health services, whether during their trajectory of displacement or in refugee-hosting countries, remains limited and challenging due to multiple factors, including language and cultural barriers and unavailability of the refugees’ health records. Cloud-based electronic health records (EHRs) are considered among the top five health technologies integrated in humanitarian crisis preparedness and response during times of conflict. This viewpoint describes the design and implementation of a scalable and innovative cloud-based EHR named Sijilli, which targets refugees in low-resource settings. This paper discusses this solution compared with other similar practices, shedding light on its potential for scalability.

## Introduction

### Global Refugee Crisis

The number of forcibly displaced individuals, including refugees, has reached an alarming rate globally, with 70.8 million forcibly displaced individuals, including around 26 million refugees [1], of whom the majority are of Syrian nationality [2,3]. More than 80% of these refugees have been internally displaced or have fled to Syria’s neighboring countries [4]. Notably, low- and middle-income countries (LMICs) host the highest number of refugees [4], with inconsistent percentages reported [5]. Figures from 2016 indicate that a total of 5 million registered Syrian refugees have fled to Syria’s neighboring countries, namely Lebanon, Jordan, Egypt, Turkey, and Iraq [6].

### Health Needs of Refugees

Refugees are often exposed to stressful conditions of vulnerability, poverty, poor nutrition, and marginalization prior to and during the journey of migration, a situation that increases their susceptibility of developing different health problems, including mental health problems [7-9]. From a health perspective, women, children, and older adults remain the most affected during the journey of migration [9]. Malnutrition, anemia, communicable and noncommunicable diseases (NCDs), women’s health, and mental health emerge as the top priority health concerns of refugees [10]. However, several barriers impede refugees’ access to the appropriate health services targeting these needs in the host countries. Examples of barriers include inadequate knowledge about the availability of health services, insufficient financial capacities, limited access to transportation, cultural differences, and language barriers and the scarcity of interpreters [11,12]. Legal restrictions such as issues of registration could also hinder refugees from accessing adequate health services in the host countries [12,13].

### Need for Innovative Solutions for Health System Strengthening in LMICs

The growing and continuous influx of refugees imposes challenges on the health systems of the refugee-hosting countries vis-à-vis catering to the needs of these populations and providing essential health services [9], especially because the majority of refugee-hosting countries are LMICs with already exhausted and fragile health care systems. Having said that, strengthening the fragile health systems of the refugee-hosting countries remains an urgent need to ensure equitable access to health services for refugee populations [9]. This in turn calls for the implementation of relatively low-cost, innovative, feasible, and contextualized solutions tailored to target the needs of these emotionally and physically distressed populations.

Electronic health records (EHRs) have been progressively adopted in resource-constrained settings [14] and conflict-affected areas [15] for the great promise they hold in providing coordinated care, sharing patient information among health care providers across different settings, reducing medical errors, and improving the quality and continuity of health care [16]. For instance, the use of EHRs avoids duplicate tests, prevents drug-drug interactions, and consequently improves patient care. In addition, EHRs allow patients’ access to their health records remotely and permit better management of their health [17].

EHRs have evolved in the past decade [16] and have been defined as a longitudinal systematic collection of electronic health information of individuals or populations [18,19] created in any health care delivery setting [20]. EHRs generally store patients’ health data, including demographic information, social history, health problems, active diagnoses and past medical history, diagnostic test results, medications, hospitalization information, consultant reports, immunizations, allergies, health screening study results, and progress notes [17].

A relatively recent type of EHRs is the cloud-based EHR system [21]. Cloud-based EHRs have been promoted in conflict-affected areas where the digital infrastructure, such as internet connectivity, is lacking, and they have been regarded as cost-effective methods that permit scalability and interoperability [22]. In fact, cloud-based EHRs not only track patients across time, but also permit the sharing of patients’ data among health care providers so that patients’ health care can be easily tracked and monitored [15]. In addition, cloud-based EHRs are considered among the top five health technologies integrated in humanitarian crisis preparedness and response during times of conflict [23]. The World Disasters Report 2013 stated that the use of cloud-based EHR is a successful approach for the recovery of health records in case of damage to physically held health records, a highly probable incidence in conflict settings [24]. Moreover, cloud-based EHRs have been regarded as resilient and effective tools that facilitate monitoring the health status of refugees over the long term, specifically in protracted crisis, and eventually improve the process of health care delivery [25-27].

### Existing eHealth Systems Targeting Refugee Populations

A few types of EHRs have been implemented for migrants and refugees [28] and have shown their potential to address some of the challenges facing these populations in accessing health care services [28,29], yet they also underline areas that need improvement. The United Nations Relief and Works Agency for Palestinian Refugees (UNRWA) developed a computer-based (eHealth) system to improve the quality and efficiency of health care services provided to Palestinian refugees living in UNRWA’s fields of operations, namely Lebanon, Jordan, Syria, West Bank, and Gaza [30]. The UNRWA’s eHealth system was developed and introduced in 2010 in response to the increasing workload and to the high prevalence of NCDs [30]. The system was associated with an improvement in the quality of health care and was described as a secure and cost-effective tool supporting the health care services provided by the UNRWA’s health program [30]. However, the UNRWA’s eHealth system covers only Palestinian refugees that are registered with the UNRWA and excludes millions of unregistered Palestinian refugees and all refugees of other nationalities worldwide. Adding to the drawbacks of the UNRWA’s eHealth system is its restricted use, whereby it is accessible solely at health facilities governed by UNRWA.

Similarly, the Refugee Assistance Information System (RAIS) is an EHR system developed by the United Nations High Commissioner for Refugees (UNHCR) to track, monitor, and provide assistance to refugees registered with UNHCR [25,31]. It collects basic demographic information and some health services data of UNHCR-registered refugees [25,31]. However, the RAIS remains incomprehensive regarding health data and is exclusively created for refugees registered with UNHCR, leaving behind millions of unregistered refugees.

### The Context of Lebanon

Lebanon, one of the main LMICs housing refugees, hosts around 1 million registered Syrian refugees in addition to more than 400,000 unregistered refugees [32], rendering it the country with the highest number of refugees per capita worldwide [5]. This massive influx of refugees to Lebanon, coupled with their increased demand for health services, has consequently created an unprecedented strain on the local health care system, originally characterized by its fragility [33]. The situation is further aggravated by refugees residing mainly in underserved rural Lebanese areas, where health service delivery is relatively suboptimal and insufficient [34].

Regarding the use of eHealth systems for refugees in Lebanon, both UNRWA’s eHealth system and the RAIS are applicable in the country, yet the use of both systems remains bounded by the aforementioned restrictions.

Another example of existing technologies in Lebanon is Phoenix. Phoenix is an EHR system developed by the Lebanese Ministry of Public Health (MoPH), and it functions exclusively among the 229 primary health care centers (PHCs) that operate under the supervision of the MoPH. Phoenix EHR targets disadvantaged Lebanese and refugees from different nationalities who receive primary health care within the national network of PHCs governed by the MoPH. It contains vital patient information, including documentation, guidelines, orders, and results [35]. The system allows the exchange of patient health information among health care providers within each PHC and the MoPH; however, the health information included in Phoenix EHR cannot be shared across the different PHCs belonging to the network. Instead, a new health record has to be created again whenever a patient visits a new PHC for the first time. In addition, Phoenix EHR does not function at Lebanese hospitals or mobile health clinics, where a significant proportion of refugees seek health services.

## Sijilli: A Cloud-Based EHR for Refugees in Low-Resource Settings

### Conception of Sijilli

The pressing need for innovative digital solutions that would enhance refugees’ access to needed health services in low-resource settings, regardless of their migration journey, triggered the development of Sijilli (meaning “my record” in Arabic).

This paper describes the design of the cloud-based mobile EHR system and reports on its implementation among Syrian refugees in Lebanon. Sijilli [36] was launched in 2018 as a collaboration between the Global Health Institute (GHI) at the American University of Beirut (AUB) in Beirut, Lebanon, and the health care software company Epic in the United States. Sijilli was designed in a contextualized approach to cater to refugees’ need for universally accessible EHRs and to fill the several gaps identified in existing practices and available similar technologies. It aims to securely collect and preserve essential health data for refugees during and beyond their displacement journey, besides ensuring data security, universal access, and interoperability of the health record data. Of note, more than 10,000 Syrian refugees across Lebanon currently possess a Sijilli EHR.

### The Concept Design and Implementation

Sijilli is designed to allow interaction and to support convenient workflows among different users, from the data entry personnel to the administrators and ultimately to the end users, namely health providers and refugees.

**Figure 1:**
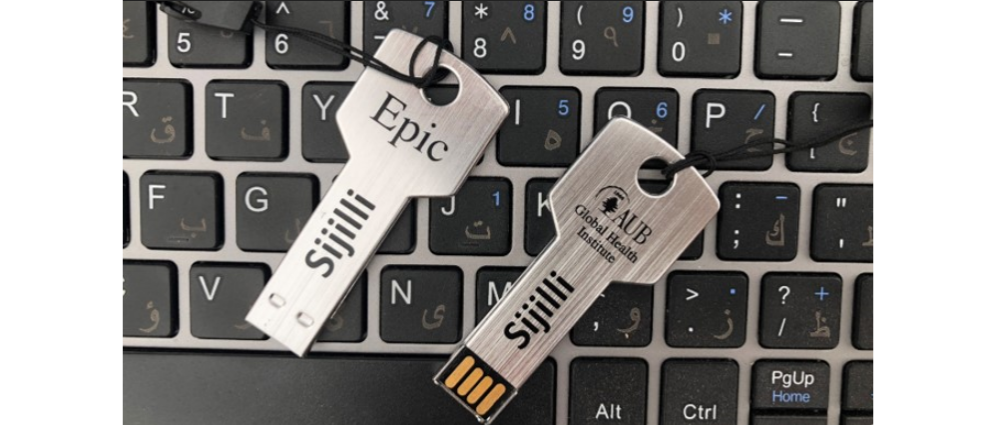
The key-shaped USB containing the Sijilli electronic health record handed to the refugee.

Data entry personnel collect the health information from refugees in different remote and underserved areas using data entry–friendly software run on tablet computers. Worth mentioning is that the use of the Sijilli software system by data entry personnel does not require internet connectivity and is done completely offline. The system adopts a simple user interface design to accommodate users and data collectors of diverse health backgrounds. Once the health information of the refugee is entered into the system, the Sijilli data collection software generates a password-protected and advanced encrypted standard (AES) PDF document of the health record of the refugee. The password of the PDF is generated exclusively and blindly (ie, without input from the data entry personnel) for the Sijilli holder and consists of the first letters of the refugee’s first name, father’s name, mother’s name, and family name, in addition to the 4 numbers of their year of birth, entered in this specific order. This simple composition of the password allows Sijilli holders to recall their passwords by following simple instructions. The Sijilli health record in its PDF version is then downloaded on a key-shaped flash drive (Figure 1) and handed to the refugee for use across different health facilities worldwide without any restrictions. In parallel, an encrypted deidentified version of the generated health record is uploaded to the Sijilli cloud-based server. The cloud-based version of the health record of any Sijilli holder can be accessed globally by either the patient or the health provider via the Sijilli website [37] following a 2-step identity verification process. The multiple layers of security (ie, USBs, hardware used for data entry, and internal and external servers of the cloud-based system) are crucial to ensure data security and privacy of the Sijilli system.

Worth noting is that patients cannot alter any data contained in their Sijilli records, neither through the PDF on the flash drive nor through the patient’s portal on the Sijilli website. On the other hand, health providers can have access to refugee patients’ Sijilli records when provided with the personal identification number (PIN) and the security questions that are recognized exclusively by Sijilli holders/refugees. External health providers can see all the health data of the refugee, originally collected by the data entry personnel at the time of the creation of the health record, and can also add clinical notes online at the time of encounter with the patient using the cloud-based version of the Sijilli EHR. No modifications to the original patient information can be made by the health providers. This ensures traceability and protects the liability of the GHI and Epic team.

### Architecture of Sijilli

#### Clinical Development

The Sijilli EHR is composed of 7 sections covering sociodemographic information, social and lifestyle habits, medical and surgical history, obstetrics and gynecological (OB/GYN) conditions for women only, medication use, vaccination history, and mental health screening. The data entered in the sociodemographic section allow the creation of the password and security questions used at times of access to the health record.

The sociodemographic section includes questions on basic sociodemographic information, such as the previous occupation in the country of origin where conflict is taking place and the current occupation in the refugee-hosting country, which allows the identification of potential occupational health hazards and risk factors. Other information collected includes the location of the settlement and the year of migration, among other information. The section also includes questions that may affect the exposure of the refugee to health risks, including parental consanguinity, given that consanguineous marriage is a deeply rooted social practice among the Syrian refugee population. Knowledge about parental consanguinity enables the health care provider to explore and identify genetic disorders such as thalassemia. Allergies are also entered in this section to indicate any source of allergy that the refugee has.

Further risk factors can be identified through data entered into the social and lifestyle section, which namely addresses smoking, alcohol drinking, and physical exercise. As the Syrian refugee crisis shifts from an acute emergency to a protracted crisis, NCDs and preventative medicine become a vital aspect of the health care response to refugee populations.

The medical and surgical history section was designed to allow the selection of one or multiple medical conditions or surgical procedures, if any, using a drop-down menu that includes the most common conditions and surgeries, derived from the International Classification of Diseases, 10th Revision developed by the World Health Organization [38]. Diseases not found in the drop-down list can be entered manually by data entry personnel. Worth noting is that the list of medical conditions and surgical procedures was constantly updated throughout the course of implementation according to the frequency of encounter of medical conditions to ensure a standardized method of data entry.

With regards to the OB/GYN section, it is worth noting that this section was not originally embedded in the system, given that OB/GYN-related conditions were included in the general medical and surgical history section. However, a stand-alone OB/GYN section was added during the project implementation phase to take into consideration that the majority of the refugees having their Sijilli records created were women of child-bearing age, many of whom were pregnant. Moreover, a high pregnancy complication rate was noted by the women, which required the addition of a section dedicated to OB/GYN that included pregnancy-related complications to ensure proper and comprehensive documentation. The aforementioned section includes questions on gravida, para, aborta, method of delivery and associated complications, breastfeeding practice, and use of contraception.

To record any medications that the refugee is taking, a section on medication use is included in the Sijilli software system. Medications included cover mainly those taken for chronic conditions, in a drop-down menu format. Categorization of medications was based on *Davis’s Drug Guide for Nurses*, which classifies medications according to their type (eg, statins, antihypertensive drugs, etc) [39]. The data entry personnel are also able to enter any other medication taken beyond those in the list. The dosage, frequency of use, and start date of use for each entered medication are mandatory fields. This section remains necessary to indicate to the health provider any potential drug-drug interactions at the time of medication prescription.

The vaccination history section was included due to the outbreak of communicable diseases among the refugee population, such as the repetitive measles outbreaks witnessed among the refugee population in Lebanon. This section includes all required vaccines by the Lebanese MoPH. It is also designed to allow data entry personnel to indicate if school vaccines were given in Syria, given that this was generally a routine practice.

The record then navigates to the mental health screening section, which uses the Patient Health Questionnaire-9 (PHQ-9). It is well established that refugees are at an increased risk of posttraumatic stress disorder, major depressive disorder (MDD), generalized anxiety disorder, and other behavioral and emotional problems. We elected to screen for MDD in particular, as previous research has shown that it is the most common and debilitating condition among Syrian refugees in Lebanon [40]. The PHQ-2 followed by the PHQ-9 were chosen as the screening tools for depression due to their ease of use, allowing for data collectors and data entry personnel with limited clinical experience.

#### Technical Development

The Sijilli EHR system was designed to give different levels of access authorization to different users, such as data collectors and data entry personnel, administrators, external health providers, and refugees. Data entry personnel are on the front lines collecting information from the refugee population in areas often lacking network connectivity. All data from the data entry hardware (eg, laptops, tablets, etc) hosting the Sijilli data collection software can be wiped out after being synced back to the hub. The administrators, on the other hand, can access the Sijilli administrator web application, which is not available outside the internal network. The application can be used to authenticate hardware used for data collection and data entry, process synced requests, and update code sets that can be used in the Sijilli data collection software. Administrators can also generate a deidentified report of the health records for research purposes. The administrator web-based application can only be used by users authenticated in the AUB network and authorized by administrators through the web application. External health providers can access a specific refugee’s Sijilli EHR by providing a PIN and answering security questions, which are only recognized by the refugee, examine a summary of the refugee patient’s Sijilli EHR, and add clinical notes for future care sought by the refugee*.* In parallel, refugees who are Sijilli holders can also access their respective record through the password-protected PDF version of the EHR or through the portal on the Sijilli website by providing their PIN and answering security questions about their record.

### Key Features of Sijilli’s Technical Architecture

**Figure 2:**
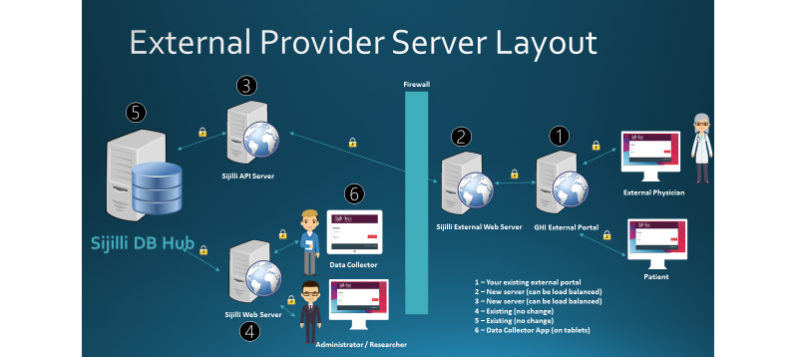
Architecture diagram of the Sijilli electronic health record. API: application programming interface; DB: database; GHI: Global Health Institute.

Figure 2 shows the technical architecture diagram of the Sijilli EHR, which is based on several key features.

#### Multilayer Security

Given that Sijilli is a cloud-based EHR, data security is of particular concern and has been considered by addressing several key points. These include access to data, which is driven by role-based user access whereby once the data get into the database, only external health providers and the refugees can access patient records by providing the PIN and answering specific security questions. The access of administrators is restricted to deidentified personal health information of the refugees following data upload to the Sijilli database. In terms of authentication, hardware used by data entry personnel are independently authenticated by the administrator. In cases where laptops are used for data collection and data entry, the disk on the Sijilli data collection application is encrypted to protect against theft. Data are then wiped out after being uploaded to the Sijilli hub database. The PDF files generated in the Sijilli data collection application are password protected with a refugee-friendly PIN and AES encryption. For access through the Sijilli portal, external health providers and refugees can only access one Sijilli record at a time, and a captcha check has been added to the external web applications to prevent robots from accessing multiple records. All data in motion were set to be network protected using transport layer security 1.2. An audit trail exists in the hub database to indicate the access patterns to the health records by external health providers.

#### Scalability

Sijilli is characterized by 3 independent server applications that are cloud enabled and can be independently scaled up based on load and usage patterns. The Sijilli data collection application can be installed on as many laptops or tablets as required for data collection/data entry, and the syncing of data occurs when the laptops or tablets used are within the internal network.

#### Extensibility and Maintainability

Administrators can manage the code sets and other metadata associated with the Sijilli application from the administrator application. This metadata can be synced to all the Sijilli data collection applications when the data from the data collector application are downloaded to the Sijilli hub. In terms of maintainability, the server teams have the authority to install the updates to the applications independently of each other.

### Integration and Contextualization

A key feature of the Sijilli EHR is its contextualized design. The design of each of the Sijilli data collection applications, the EHR itself, and the Sijilli website portals was tailored to the needs and interest of each of the multiple users/stakeholders involved.

First, the design of the Sijilli data collection application used for primary creation of the Sijilli EHR for refugees was based on the input of several health providers, including physicians, nurses, community health workers, medical residents, and medical students. This was done to ensure that the platform developed is user-friendly at the time of Sijilli EHR creation and time efficient in terms of data entry and collection. The Sijilli data collection application then underwent beta testing on a sample of 100 refugees, after which the application underwent some calibration and fine-tuning steps. These mainly included changes in the units of categories used, the addition of some medical conditions to the drop-down menu, and the addition of a complete section on OB/GYN conditions for female refugees. Health providers were also involved in the design of the Sijilli website, the portal through which they can update the cloud-based EHR of refugees visiting a health facility. This is to ensure that the website/platform is user-friendly and smooth to use. The platform includes the Arabic translation of instructions to accommodate non–English-speaking health providers, who are common in the Syrian-Lebanese context.

## Discussion

### Summary

Migrating populations and refugees continue to face obstacles in accessing health care services [41] and experience gaps in the continuity of health care [29], which differs across host countries and during the stages of the migration process, a fact that poses a negative impact on their health status [41]. One of the biggest challenges that faces refugees during and beyond their migration journey is access to medical records [41]. In view of this, EHRs seem to have the potential to address some of these challenges and provide opportunities to increase access to health care services and eventually improve the quality and continuity of health care [29]. However, implementation of EHRs in LMICs is still limited [42].

Previous studies have examined the association between EHR adoption and improvement in the quality and continuity of health care in refugee settings and have emphasized the importance of the adoption of these innovative technologies for the great promise they hold in such resource-constrained settings [26,30,43-46]. A series of cohort studies by Khader et al [26,43-46] evaluated the effectiveness of the implementation of an electronic medical record (EMR) cohort monitoring system to follow up on Palestinian refugees with hypertension [26,44] and diabetes [43,45,47] in Jordan. Despite minimal challenges encountered, mainly operational challenges [47], the authors reported promising results and concluded that an EMR cohort monitoring system is an efficient tool in a refugee context in terms of management and follow-up of NCDs [26,43-46].

### Comparison With Similar eHealth Practices

The previously described UNRWA’s eHealth system [30] and the RAIS [25,31,47] have been considered simple, secure, and cost-effective tools that have been correlated with an improvement in the quality and continuity of health care among refugee populations. Although they both collect some health information, Sijilli EHR appears to be more comprehensive compared with the UNRWA’s eHealth system and the RAIS, wherein it covers a more inclusive range of health data, including context-specific components, such as parental consanguinity and mental health, which are not covered by either of the other systems. For instance, consanguineous marriage, a common practice among refugees [48], is well known to be a risk factor for a variety of genetic diseases [49], while mental health disorders have been reportedly high among refugee populations [50]. Therefore, such context-specific components permit better evaluation and monitoring of the health status of refugees during their migration journey and beyond.

In terms of inclusiveness, the UNRWA’s eHealth system is designed to target Palestinian refugees that are registered with the UNRWA, and it is only functional through the health centers in the 5 fields of UNRWA’s operations [30]. Likewise, the RAIS is exclusively created for refugees registered with the UNHCR [25,31,47], and only UNHCR officials have access to the health record data [31]. Accordingly, both systems exclude millions of unregistered refugees worldwide. Conversely, although initially created for Syrian refugees across Lebanon, Sijilli is designed to take into consideration the potential for upscaling to cover refugees of any nationality worldwide and to provide them and their health care providers with global access to their health records wherever the migration journey takes them.

### Potential for Upscaling

With the impending challenge of refugee migration, initially to neighboring host LMICs, followed by opportunistic migration to European and possibly North American countries, the importance of an innovative, universally accessible, and digital technology becomes more emphasized [23]. According to asylum-seeker data from UNHCR on refugee migration and resettlement [51], more than half a million Syrians moved to Germany seeking asylum, approximately 100,000 moved to Sweden, and 50,000 moved to Austria. Additionally, about 100,000 have moved to North America, indicating the desire of refugees to seek final settlement in developed countries. Even beyond the example of Syrian refugees, the world is witnessing many other migration crises, such as the hundreds of thousands of Rohingya fleeing from Myanmar to Bangladesh and a similar number of Venezuelans seeking asylum in the United States, among others [52]. eHealth and EHRs are well known in developed countries and have proven to be impactful in enhancing the quality of clinical care. The obstacle to scaling such solutions to LMICs has been the limiting high costs to purchase and maintain such solutions and therefore, the development of low-cost cloud-based tools offers the needed solutions to support access and provision of health care to vulnerable populations [53]. Literature indicates that health care providers and governments are keen to improve access to, engagement with, and delivery of health care through the use of eHealth [23]. A recent World Bank Report highlighted that many donor agencies are currently focusing on scalable eHealth interventions for LMICs that are low cost and mobile, mainly to resolve the barriers of access [53]. Such innovative solutions can also account for issues related to continuity of care and language barriers, while providing users with adequate health services.

Sijilli’s potential for scalability and interoperability lies in the fact that Sijilli is a low-cost cloud-based EHR that can be easily exported to conflict-affected or crisis-affected areas that lack the required digital infrastructure, and, more importantly, it omits the need for physically held patient records. Furthermore, such solutions can be transported with the migrating user across several host settings with guaranteed security that puts the refugee and the host service provider at ease. Countries with fragmented health care systems would also benefit from a parallel solution, which would allow service providers to exchange and consolidate the patient’s health-related information and interventions in one comprehensive, secure, and easy-to-access EHR.

### Conclusions

In conclusion, the Sijilli EHR represents a model for an innovative and scalable cloud-based solution that could be replicated for several vulnerable populations in different low-resource or crisis settings. Sijilli EHR's contextualized design, architecture with multilayered security, and process design and implementation highlight the potential applicability of this digital health solution to different populations and settings, with specific considerations for cases of different contexts and users. Sijilli EHR has a crucial role in enhancing refugees’ access to health services throughout the displacement and migration journey.

## Abbreviations

AES: advanced encrypted standard
AUB: American University of Beirut
EHR: electronic health record
EMR: electronic medical record
GHI: Global Health Institute
LMIC: low- and middle-income countries
MDD: major depressive disorder
MoPH: Ministry of Public Health
NCD: noncommunicable disease
OB/GYN: obstetrics and gynecology
PHC: primary health care center
PHQ: Patient Health Questionnaire
PIN: personal identification number
RAIS: Refugee Assistance Information System
UNHCR: United Nations High Commissioner for Refugees
UNRWA: United Nations Relief and Works Agency for Palestinian Refugees
